# Self-perception of dental aesthetics and social media influence among students at a Palestinian dental school

**DOI:** 10.1038/s41405-026-00445-w

**Published:** 2026-05-19

**Authors:** Naji Ziad Arandi, Fatima Qtait

**Affiliations:** 1 Department of Conservative Dentistry, Faculty of Dentistry, Arab American University, Jenin, Palestine; 2 Undergraduate Dental Student (5th Year), Faculty of Dentistry, Arab American University, Jenin, Palestine

**Keywords:** Dental education, Aesthetic dentistry

## Abstract

**Aim:**

This study aimed to evaluate dental students’ self-perception of dental aesthetics, aesthetic treatment preferences, and the perceived influence of social media and to examine whether these outcomes differed by sex and training stage.

**Methods:**

An online cross-sectional survey was conducted among undergraduate dental students at the Faculty of Dentistry, Arab American University, Palestine. A total of 246 students were selected via stratified random sampling by academic year and completed a structured questionnaire. Subgroup analyses were performed by sex and training stage.

**Results:**

Composite resin was the preferred restorative material (87.4%), and natural white (A1–A2) was the most preferred anterior shade (79.3%). Teeth whitening was the most common aesthetic treatment (40.2%). Instagram was the main platform for viewing aesthetic dental content (80.5%); 70.7% reported a moderate-to-very strong social media influence on their ideal smile, and 47.2% considered an aesthetic procedure after exposure to social media content. In subgroup analyses, female participants reported greater dissatisfaction with gingival display. Clinical-stage students more often notice gingival defects and are more likely to judge social media-driven aesthetic outcomes as not clinically achievable under routine practice conditions.

**Conclusion:**

Dental students reported generally positive self-perceptions and strong preferences for natural, tooth-coloured aesthetics, alongside high exposure to social media and substantial perceived influence. Differences by sex and training stage suggest that aesthetic awareness and expectations may evolve with clinical experience. These findings highlight the importance of supporting students in critically evaluating online aesthetic content and communicating clinically feasible outcomes.

## Introduction

Smile aesthetics are an important aspect of facial appearance and may influence social interaction, perceived attractiveness, and interpersonal judgments [1, 2]. As dentofacial aesthetics have become increasingly important in both social and clinical contexts, greater attention has been given to tooth colour, alignment, and overall smile harmony, contributing to increased demand for aesthetic dental care and heightened awareness of ideal smile characteristics [3, 4]. In dentistry, smile assessment is based on established criteria related to the face, lips, gingiva, and teeth [2]. These assessments inform treatment planning, patient communication, and expectation management [5]. In undergraduate dental education, aesthetic concepts are introduced across multiple courses and become more prominent during the clinical years as students begin applying aesthetic principles in patient care [5, 6]. Dental students’ perceptions of dental aesthetics are therefore relevant because they may influence their clinical interests, treatment preferences, and communication with patients [7–11]. Social media has become an important influence on aesthetic perceptions and expectations. Platforms such as Instagram, TikTok, and Snapchat commonly present curated and digitally enhanced images that may promote narrow or unrealistic standards of smile aesthetics [12–15]. Exposure to such content may shape how young adults evaluate dental appearance, interpret aesthetic norms, and perceive the desirability or feasibility of cosmetic dental procedures. This influence may be especially relevant among dental students, whose views are shaped by both personal exposure and professional training.

Evidence from Palestine remains limited regarding how dental students perceive their dental aesthetics, which aesthetic treatments and shades they prefer, and whether social media influences these perceptions and expectations. It is also unclear whether these outcomes differ by sex and training stage. These findings may help inform undergraduate dental education, particularly in relation to aesthetic teaching, critical appraisal of social media content, and communication of realistic treatment outcomes.

This study assessed dental students’ aesthetic self-perception, aesthetic preferences, perceived social media influence, and professional perceptions related to dental aesthetics and examined differences by sex and training stage.

## Methods

### Study design and setting

This cross-sectional survey was conducted among undergraduate dental students at the Faculty of Dentistry, Arab American University, Palestine, from November 2025 to January 2026. The study was reported in accordance with the Strengthening the Reporting of Observational Studies in Epidemiology (STROBE) guidelines for cross-sectional studies.

### Study population and sampling strategy

The official student enrolment list served as the sampling frame. All the undergraduate dental students enroled at the Faculty of Dentistry, Arab American University (*N* = 642), were eligible to participate. A minimum sample size of 246 students was calculated via a single-population proportion formula with finite population correction, assuming a 95% confidence level, a 5% margin of error, and an expected proportion of 50%, as no prior local estimate was available for the main outcomes. The 50% assumption was used because it yields the most conservative sample size estimate.

Stratified random sampling with proportional allocation by academic year (1st to 5th year) was used. This ensured proportional representation across training levels and reduced the risk of overrepresenting any single group. Stratification by academic year was appropriate because educational exposure and clinical experience vary by training stage and are directly relevant to the study outcomes. For analysis, academic years were further grouped into preclinical (1st–3rd years) and clinical (4th–5th years) training stages.

**Fig. 1:**
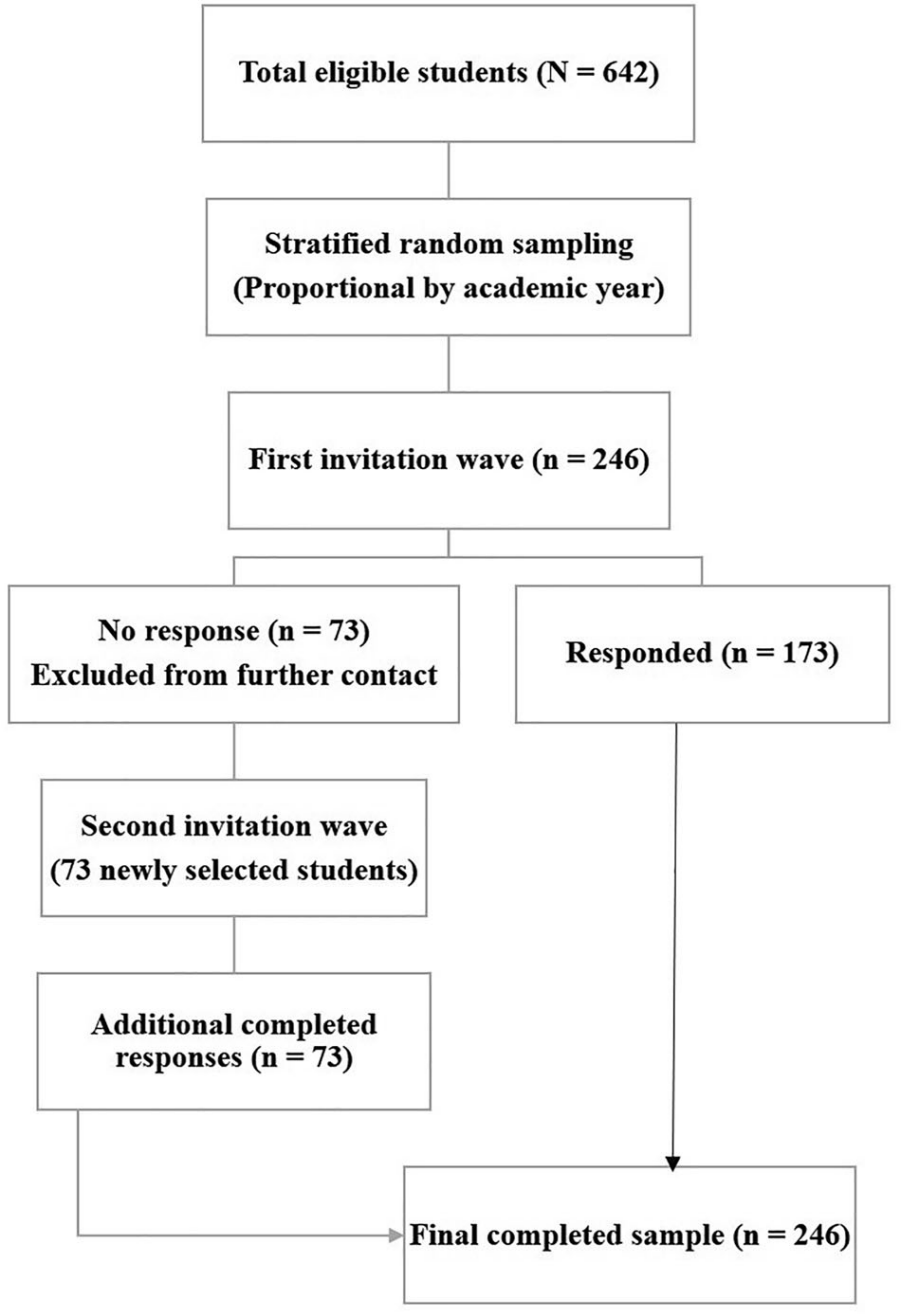
Flowchart of the sampling and recruitment process for undergraduate dental students.

Invitations were distributed through official class social media groups, with up to two reminders sent at 2-week intervals. In the first wave, 246 students were invited, of whom 173 completed the survey and 73 did not respond. These nonresponders were excluded from further recruitment, and 73 newly selected students from the same sampling frame were invited in a second wave to achieve the target sample size. To restrict access to invited students and prevent duplicate responses, participants were required to use their institutional student email before completing the questionnaire. All questionnaire items were mandatory, resulting in complete item-level data with no missing responses. A flowchart of the sampling and recruitment process is shown in Fig. 1.

### Questionnaire development and validation

Data were collected via a structured online questionnaire administered via Google Forms, with items adapted from previously published studies [9, 15]. Face and content validity were assessed by two faculty members with expertise in aesthetic dentistry and dental public health. The questionnaire was pilot tested on 30 dental students not included in the final analysis to assess clarity and comprehension. Minor revisions were made to simplify wording and clarify selected response options, particularly for the social media items. Because the questionnaire covered multiple distinct domains rather than a single underlying construct, formal psychometric validation and internal consistency testing were not performed.

The final questionnaire consisted of five sections covering demographic and academic characteristics, self-perception of dental aesthetics, preferred aesthetic treatments and shade/restorative material preferences, exposure to aesthetic dental content on social media and its perceived influence, and professional aspects related to dental aesthetics. All the questionnaire items are provided in Appendix 1.

### Statistical analysis

Statistical analyses were conducted in IBM SPSS Statistics v29 (IBM Corp.). The variables were categorical or ordinal and are summarised as n (%). Associations were tested using Pearson’s chi-square test, with Fisher’s exact test for 2 × 2 tables when the expected cell counts were small. Subgroup analyses were prespecified for sex and training stage (preclinical: 1st–3rd years; clinical: 4th–5th years). The prespecified primary outcomes were noticing gingival defects when smiling (B4), perceiving tooth display as nonideal (B8), perceived social media influence on the ideal smile (D3; moderate–very strong vs none/slight), and perceived feasibility of social-media aesthetic outcomes under routine clinical practice conditions (D6; no vs yes). Inference focused on these outcomes, whereas other item-level comparisons were treated as exploratory. Formal adjustment for multiple testing was not applied to the nonprespecified comparisons because the questionnaire items were interrelated, and strict correction methods (e.g., Bonferroni) may be overly conservative in this context. These findings should therefore be interpreted cautiously.

For subgroup comparisons, crude ORs with 95% CIs were calculated from 2 × 2 tables. The frequency items were dichotomised as at least sometimes (sometimes/often/very often) versus rarely/never, and infrequent response options were collapsed (e.g., ‘Other’) to limit sparse cells. This approach was used selectively to support clinically interpretable subgroup comparisons and logistic regression analyses based on sufficiently populated categories. Binary logistic regression models were fitted for selected prespecified outcomes to estimate adjusted odds ratios (aORs) with 95% confidence intervals, adjusting for sex and training stage, which were the prespecified subgroup variables of interest. Other potentially relevant confounders were not comprehensively measured and therefore were not included in the models. The results are presented for the comparison of interest (training stage adjusted for sex or sex adjusted for training stage). For item D6, the event was coded as 1 = ‘No’ (not feasible) and 0 = ‘Yes’ (feasible).

### Ethical considerations

Ethical approval was obtained from the Arab American University Institutional Review Board (Approval No. J-2025/A/86/N). Participation was voluntary, and electronic informed consent was obtained before questionnaire access. The students were informed that nonparticipation carried no academic penalty. The responses were anonymized before analysis in accordance with the Declaration of Helsinki.

## Results

Table 1 presents the demographic and academic characteristics of the participants. Among the 642 eligible undergraduate dental students, 319 were invited, and 246 completed the survey. The participants were predominantly female (72.8%), and all questionnaire items were completed.

**Table 1:** Demographic and academic characteristics of participants (*n* = 246)

Academic year	Training stage	Total n (%)	Male *n* (%)	Female *n* (%)
1st	Preclinical	23 (9.3)	3 (13.0)	20 (87.0)
2nd	Preclinical	28 (11.4)	4 (14.3)	24 (85.7)
3rd	Preclinical	42 (17.1)	16 (38.1)	26 (61.9)
4th	Clinical	57 (23.2)	18 (31.6)	39 (68.4)
5th	Clinical	96 (39.0)	26 (27.1)	70 (72.9)

Values are *n* (%). Percentages are calculated out of *n *= 246.

Overall, the participants reported generally favourable self-perceptions of dental aesthetics (Table 2). Satisfaction was highest for gingival appearance, tooth shape, and tooth colour, whereas smaller proportions reported concerns related to tooth display or gingival display. In prespecified subgroup analyses, clinical-stage students were more likely than preclinical students to report noticing gingival defects when smiling and to perceive their tooth display as nonideal. Compared with male participants, female participants were more likely to report dissatisfaction with the amount of gingival display. The detailed item-level results are shown in Table 2. Academic-year patterns are reported descriptively (exploratory); detailed academic-year distributions by sex are provided in Appendix 2. Unadjusted ORs were derived from 2 × 2 comparisons; adjusted ORs were estimated via logistic regression.

**Table 2:** Self-perception of dental aesthetics and routine dental attendance

Question/Item	Yes *n* (%)	No *n* (%)	*p* value (Sex)	*p* value (Training stage)
B1. Prefer selfies from a particular side	186 (75.6)	60 (24.4)	0.135	0.447
B2. Wish smile looked like those in media	119 (48.4)	127 (51.6)	0.775	0.602
B3. Pay particular attention to others’ teeth/smile	234 (95.1)	12 (4.9)	0.094	0.062
B4. Notice gum defects when smiling in mirror	146 (59.3)	100 (40.7)	0.467	0.033*
B5. Notice tooth defects when smiling in mirror	42 (17.1)	204 (82.9)	0.253	0.222
B6. Satisfied with tooth colour	165 (67.1)	81 (32.9)	0.547	0.401
B7. Satisfied with gum appearance	213 (86.6)	33 (13.4)	1.000	0.441
B8. Tooth display in smile is not ideal	53 (21.5)	193 (78.5)	0.487	0.011*
B9. Dissatisfied with amount of gum show	48 (19.5)	198 (80.5)	0.011*	0.324
B10. Teeth too long/too short	36 (14.6)	210 (85.4)	0.547	0.097
B11. Teeth too wide/too narrow	36 (14.6)	210 (85.4)	0.547	1.000
B12. Satisfied with tooth shape	198 (80.5)	48 (19.5)	0.588	0.135
B13. Regularly visit dentist for routine care	132 (53.7)	114 (46.3)	0.196	0.147

Values are *n* (%). The percentages are based on n = 246. *p* values were calculated using Pearson’s chi-square test or Fisher’s exact test, as appropriate. Statistically significant *p* values (*p* < 0.05) are indicated by bold text and an asterisk *.

Aesthetic preferences are summarised in Table 3. Composite resin was the preferred posterior restorative material, natural white shades (A1–A2) were the most frequently preferred anterior shade, and tooth whitening was the most commonly preferred aesthetic treatment. According to the subgroup analyses, clinical-stage students were more likely than preclinical students to prefer composites for posterior restorations and A1–A2 shades for anterior teeth. Personal treatment preference differed by sex, whereas perceived knowledge of common aesthetic treatments did not differ significantly by sex or training stage.

**Table 3:** Aesthetic treatment and shade preferences

Question/Item	Response categories, n (%)	*p* value (Sex)	*p* value (Training stage)
C1. Sufficient knowledge about common aesthetic treatments	Yes: 136 (55.3)	0.472	0.186
	No: 110 (44.7)		
C2. Preferred material for a posterior restoration	Composite: 215 (87.4)	0.521	<0.001*
	Amalgam: 21 (8.5)		
	GIC/Bioactive: 10 (4.1)		
C3. Aesthetic treatment personally preferred	Teeth whitening: 99 (40.2)	0.046*	0.756
	Orthodontic treatment: 59 (24.0)		
	No treatment needed: 54 (22.0)		
	Other options*: 34 (13.8)		
C4. Most aesthetic shade for anterior teeth	Natural white (A1–A2): 195 (79.3)	0.292	<0.001*
	Moderate white (A3–B2): 35 (14.2)		
	Bright white (B1–BL1): 8 (3.3)		
	No preference: 6 (2.4)		
	Darker/yellowish (A3.5–B3): 2 (0.8)		

Values are *n* (%). The percentages are based on *n* = 246. *p* values were calculated using Pearson’s chi-square test or Fisher’s exact test, as appropriate. For hypothesis testing, C2 was analysed as a composite versus other materials, and C4 was analysed as A1–A2 versus other shades. Statistically significant *p* values (*p* < 0.05) are indicated by bold text and an asterisk *.

The level of exposure to dental aesthetic content on social media was high (Table 4). Instagram was the most commonly reported platform, and most participants reported viewing aesthetic dental content at least sometimes. Social media was perceived to influence the idea of an ideal smile in a substantial proportion of participants, and nearly half reported having considered an aesthetic procedure because of content seen online. Compared with preclinical students, clinical-stage students were more likely to report frequent viewing of aesthetic dental content, search online for related information or tutorials, and judge social-media aesthetic outcomes as not clinically achievable under routine practice conditions. No significant subgroup differences were observed for perceived influence on the ideal smile.

**Table 4:** Social media exposure and related perceptions

Question/Item	Responses, *n* (%)	*p* value (Sex)	*p* value (Training stage)
D1. Main platform used for viewing dental/aesthetic content	Instagram: 198 (80.5)	0.021*	0.072
	TikTok: 27 (11.0)		
	YouTube: 10 (4.1)		
	Facebook: 9 (3.7)		
	Other: 2 (0.8)		
D2. Frequency of viewing dental/aesthetic content	Never: 2 (0.8)	0.313	0.011*
	Rarely: 13 (5.3)		
	Sometimes: 110 (44.7)		
	Often: 87 (35.4)		
	Very often: 34 (13.8)		
D3. Social media influence on the idea of an ideal smile	Not at all: 27 (11.0)	0.578	0.121
	Slightly: 45 (18.3)		
	Moderately: 95 (38.6)		
	Strongly: 65 (26.4)		
	Very strongly: 14 (5.7)		
D4. Considered an aesthetic procedure due to social media	Yes: 116 (47.2)	1.000	0.357
	No: 130 (52.8)		
D5. Searched online for information/tutorials due to online content	Yes: 130 (52.8)	0.566	0.032*
	No: 116 (47.2)		
D6. Perceived feasibility of social-media aesthetic outcomes under routine clinical practice conditions	Yes: 157 (63.8)	0.373	<0.001*
	No: 89 (36.2)		
D7. Social media trends affect personal aesthetic expectations	Not at all: 29 (11.8)	0.340	0.056
	Slightly: 52 (21.1)		
	Moderately: 94 (38.2)		
	Greatly: 57 (23.2)		
	Extremely: 14 (5.7)		
D8. Want more aesthetic dentistry emphasis in education	Yes: 202 (82.1)	0.091	0.125
	No: 44 (17.9)		
D9. Trustworthiness of social media aesthetic dental content	Not at all: 4 (1.6)	0.417	0.223
	Slightly: 49 (19.9)		
	Moderately: 152 (61.8)		
	Very: 36 (14.6)		
	Extremely: 5 (2.0)		

Values are *n* (%). Percentages are calculated out of *n* = 246. Statistically significant *p* values (*p* < 0.05) are indicated by bold text and an asterisk *. *P* values comparing sex and preclinical vs clinical groups were computed via Pearson’s chi-square test for multicategory items and Fisher’s exact test for 2 × 2 comparisons (or, when expected, small cell counts). For the stage-comparison OR reported in the text, D2 was dichotomized as ‘sometimes or more frequently’ vs ‘rarely/never.’ To avoid sparse cells, infrequently selected response options were collapsed into “Other” or omitted when zero.

Professional perceptions related to dental aesthetics are presented in Table 5. Most participants believed that a dentist’s own smile or facial appearance influences patient trust, and more than half indicated that they would consider an aesthetic dental procedure to enhance their professional image. Training-stage differences were observed for how often participants were asked for opinions on “ideal smiles” and for confidence in explaining the difference between clinically feasible outcomes and digitally enhanced presentations, whereas no significant sex differences were observed for Section E items.

**Table 5:** Professional perceptions related to dental aesthetics

Question/Item	Responses, *n* (%)	*p* value (Sex)	*p* value (Training stage)
E1. Dentist’s smile influences patient trust	Yes: 238 (96.7)	0.453	0.714
	No: 8 (3.3)		
E2. Consider aesthetic treatment to enhance professional image	Yes: 139 (56.5)	0.885	0.596
	No: 107 (43.5)		
E3. How often others ask your opinion on “ideal smiles”	Never: 28 (11.4)	0.245	0.026*
	Rarely: 40 (16.3)		
	Sometimes: 114 (46.3)		
	Often: 45 (18.3)		
	Very often: 19 (7.7)		
E4. Confident explaining feasible outcomes vs enhanced presentation	Yes: 176 (71.5)	0.754	0.030*
	No: 70 (28.5)		
E5. Extent dissatisfaction affects social/academic/professional confidence	Not at all: 109 (44.3)	0.120	0.930
	Slightly: 63 (25.6)		
	Moderately: 44 (17.9)		
	Strongly: 18 (7.3)		
	Very strongly: 12 (4.9)		
E6. Would undergo aesthetic treatment if cost not a barrier	Yes: 116 (47.2)	0.200	0.693
	No: 130 (52.8)		

Values are *n* (%). Percentages are calculated out of *n* = 246. Statistically significant *p* values (*p* < 0.05) are indicated by bold text and an asterisk *. *P* values comparing sex and preclinical vs clinical data were computed via Pearson’s chi-square test for multicategory items and Fisher’s exact test for 2 × 2 comparisons (or, when expected, small cell counts).

The adjusted logistic regression models for the selected outcomes are shown in Table 6. After adjustment for sex and training stage, clinical-stage students had greater odds of noticing gingival defects when smiling, perceiving tooth display as nonideal, and judging social-media aesthetic outcomes as not clinically achievable under routine clinical practice conditions. The female participants were more likely to be dissatisfied with the amount of gingival display. The full regression outputs are provided in Appendix 3. Academic-year patterns are presented descriptively as exploratory findings (Appendix 2).

**Table 6:** Adjusted logistic regression models for selected outcomes by training stage and sex (*n* = 246)

Outcome (dependent variable)	Predictor (reference)	aOR	95% CI	p value
B4. Notice gum defects when smiling (event = Yes)	Clinical vs preclinical (ref: preclinical)	1.82	1.07–3.08	0.026*
B8. Tooth display is not ideal (event = Yes)	Clinical vs preclinical (ref: preclinical)	2.51	1.24–5.09	0.011*
B9. Dissatisfied with amount of gum show (event = Yes)	Female vs male (ref: male)	3.19	1.29–7.93	0.012*
D6. Judged social-media aesthetic outcomes as not clinically achievable under routine clinical practice conditions (event = Not achievable)	Clinical vs preclinical (ref: preclinical)	2.80	1.56–5.01	<0.001*

Binary logistic regression models were fitted for selected outcomes. aORs with 95% confidence intervals (CIs) are reported. Each model included training stage (clinical vs preclinical) and sex (female vs male) as covariates; the table presents the comparison of interest (training stage adjusted for sex or sex adjusted for the training stage). For D6, the modelled event was not clinically achievable under routine clinical practice conditions. B9 is reported to be exploratory due to its clinical relevance. All tests were two-sided, with statistical significance set at *p* < 0.05. Statistically significant *p* values (*p* < 0.05) are indicated by bold text and an asterisk *.

## Discussion

This cross-sectional survey provides one of the first datasets from a Palestinian dental school on dental students’ aesthetic self-perception, aesthetic preferences, perceived social media influence, and professional image perceptions. Overall, the findings suggest that students generally combine positive aesthetic self-perceptions with strong preferences for natural, tooth-coloured outcomes and substantial exposure to online aesthetic content. These patterns provide a useful baseline for understanding how aesthetic awareness, professional training, and digital media exposure intersect during undergraduate dental education.

Satisfaction was highest for gingival appearance, tooth colour, and tooth shape, yet specific concerns remained, particularly gingival defects, gingival display, and nonideal tooth display. This pattern supports previous findings that dental students may report overall satisfaction while still identifying specific smile-related concerns [7, 16–20]. The high proportion of students who reported paying attention to others’ teeth or smiles suggests strong aesthetic awareness, likely shaped by both personal appearance concerns and the development of professional observation. Clinical-stage students reported more detailed smile-related concerns, which may reflect more refined clinical judgments rather than lower overall satisfaction. This finding agrees with studies showing more detailed and clinically oriented aesthetic perceptions among students in advanced clinical years [5, 18, 21, 22]. Dissatisfaction with gingival display also differed by sex, suggesting that some smile-related concerns may extend beyond training-related factors. These findings suggest that aesthetic awareness does not necessarily translate into preventive behaviours, as only just over half of the students reported regular dental attendance, highlighting a persistent awareness–behaviour gap [16, 23].

The students preferred composite resin for posterior restorations, natural white anterior shades, and generally conservative aesthetic options, with teeth whitening being the most commonly preferred treatment. The greater preference among clinical-stage students for composite restorations and natural white shades may reflect cumulative curricular exposure and greater involvement in aesthetic decision-making. These findings are consistent with studies reporting a strong preference for composite over amalgam and increasing emphasis on aesthetic outcomes among both students and practitioners [9, 24–27]. The preference for whitening is also consistent with studies among Saudi and Turkish dental students, where whitening was among the most desirable aesthetic procedures [28, 29].

In the present study, high exposure to aesthetic dental content, particularly on Instagram, suggests that social media may shape students’ perceptions of ideal smile characteristics and aesthetic treatment outcomes. This aligns with recent studies showing that social media is associated with aesthetic preferences, treatment interest, and expectations regarding dental appearance [12–15]. A recent study among Turkish dental students similarly reported that social media and professional feedback influence smile perceptions, particularly among female students [29]. The perceptions of the feasibility of social-media aesthetic outcomes under routine clinical conditions were mixed, with clinical-stage students being more likely to judge such outcomes as not clinically achievable. These findings suggest that clinical experience may help students distinguish between visually appealing online portrayals and outcomes that are biologically and technically achievable in routine practice. This may reflect greater awareness of case selection, periodontal and restorative limitations, material behaviour, and patient-specific constraints [29–32].

Most students believed that a dentist’s smile and facial appearance influence patient trust, and more than half indicated that they would consider aesthetic treatment to enhance their professional image. These findings are consistent with previous literature suggesting that clinician appearance may affect perceived credibility and professionalism [33–35]. In the context of high social media exposure, this may also reflect the growing link between professional image, visual presentation, and perceived clinical authority.

Clinical-stage students were more often asked for opinions on “ideal smiles” and were more confident in explaining the difference between clinically feasible outcomes and digitally enhanced presentations. This may reflect the gradual development of professional identity, perceived authority, and communication skills during clinical training. Together with their more critical judgments of social-media aesthetic outcomes, these findings suggest that clinical exposure may help students translate aesthetic knowledge into more realistic patient communication. Although many students reported dissatisfaction with aspects of their smile, most indicated that this had little or no impact on their social, academic, or professional confidence. This contrasts with previous studies reporting greater psychosocial effects of dental appearance on confidence and social interactions, possibly because of differences in study populations and assessment methods [10, 35]. The relatively low reported impact may also reflect the use of a single self-reported item, which may underestimate subtle psychosocial effects.

Given the high exposure to and perceived influence of social media, the findings of the present study emphasise the need to teach students to critically appraise online aesthetic dental content. Recent evidence supports incorporating psychosocial awareness, media literacy, and patient-centred communication into dental education to help students manage digitally driven aesthetic expectations, appearance-based comparisons, and potential negative psychological effects [29, 36]. This may strengthen their ability to communicate realistic, patient-specific outcomes and distinguish between idealised digital portrayals and clinically achievable results. This need aligns with broader efforts in dental education to expand undergraduate exposure to aesthetic planning and digital tools that support more clinically grounded treatment communication [37, 38].

Consistent with this direction, Zotti et al. proposed and evaluated a brief undergraduate digital aesthetic dentistry course covering dental photography, digital aesthetic analysis, and digitally planned rehabilitations via accessible software (PowerPoint/Keynote) [39]. They reported that video tutorial–based delivery was better received and associated with better performance than slide-based teaching for software-dependent procedures, supporting structured, video-supported digital modules as a complement to conventional instruction [39].

### Strengths and limitations

This study has several strengths. It provides the first data from a Palestinian dental school on dental students’ aesthetic perceptions and social media influence. The use of stratified sampling ensured representation across academic years, and the study examined multiple related domains, including self-perception, preferences, and professional perceptions, within a single analytical framework.

The study was conducted in a single dental school, which limits its external validity and generalisability beyond this institutional context. The findings should therefore be interpreted as an institutional baseline rather than as nationally representative estimates. External validity may be limited by cultural differences, regional variations in social media, and differences in aesthetic dentistry training.

Recruitment across invitation waves may have introduced selection and nonresponse bias, particularly if students with greater interest in aesthetics or social media were more likely to participate. Distribution through official class social media groups may also favour participation by more digitally engaged students. In addition, the predominance of female participants may have influenced the overall prevalence estimates and should be considered when interpreting sex-related findings. The outcomes were self-reported and may therefore have been affected by recall and social desirability bias. Although the questionnaire underwent face and content review and was pilot tested before administration, it was not formally psychometrically validated. Forced responses may also have increased satisficing and introduced nondifferential misclassification (random response error), which would be expected to attenuate subgroup associations toward the null.

In addition, collapsing sparse categories and dichotomising some variables may have reduced precision and obscured more nuanced differences. The regression models were limited to sex and training stage, which were the prespecified subgroup variables of interest. Other potentially relevant confounders, such as prior orthodontic or aesthetic treatment, objective oral conditions, socioeconomic factors, and the intensity or nature of social media exposure, were not comprehensively measured and therefore could not be included. Accordingly, the adjusted estimates should be interpreted cautiously. Multiple item-level subgroup comparisons were performed without formal adjustment for multiple testing; therefore, statistically significant findings outside the prespecified outcomes should be interpreted as exploratory and hypothesis-generating.

## Conclusion

Palestinian dental students reported generally favourable self-perceptions of dental aesthetics, alongside substantial exposure to and perceived influence of social media. These findings indicate associations that should be interpreted cautiously, particularly given the cross-sectional design, self-reported measures, and single-institution setting. The findings support the reinforcement of critical appraisal of online aesthetic information and the communication of clinically feasible outcomes in undergraduate training.

## Supplementary information

Appendix 1

Appendix 2

Appendix 3

## Data Availability

The datasets of the study are available from the corresponding author on reasonable request.
