# Supplementary material for: Self-perception of dental aesthetics and social media influence among students at a Palestinian dental school
Source: BDJ Open. 2026 May 19;12:53. doi: 10.1038/s41405-026-00445-w (PMC13184236; doi:10.1038/s41405-026-00445-w)
Supplement: Supplementary file 1 — Appendix 1 [file 41405_2026_445_MOESM1_ESM.docx]

## Dental Aesthetics, Preferences, and Social Media Influence among Dental Students in a Palestinian Dental School: A Cross-Sectional Survey

## Section A: Participant characteristics

1. Gender: □ Male □ Female

2. Academic year: □ 1st □ 2nd □ 3rd □ 4th □ 5th

## Section B: Self-Perception of Dental Aesthetics

1. Do you prefer taking selfies from a particular side because you feel your smile looks better from that angle? □ Yes □ No
2. Do you wish your smile looked like the ones you see in media? □ Yes □ No
3. When looking at others, do you pay particular attention to their teeth or smile appearance? □ Yes □ No
4. Do you notice defects in your gums when smiling in the mirror? □ Yes □ No
5. Do you notice defects in your teeth when smiling in the mirror? □ Yes □ No
6. Are you satisfied with your teeth color? □ Yes □ No
7. Are you satisfied with the appearance of your gums? □ Yes □ No
8. Do you feel the amount of tooth display in your smile is not ideal for you? □ Yes □ No
9. Are you dissatisfied with the amount of gum that shows when you smile? □ Yes □ No
10. Do you feel your teeth are too long or too short? □ Yes □ No
11. Do you feel your teeth are too wide or too narrow? □ Yes □ No
12. Are you satisfied with the shape of your teeth? □ Yes □ No
13. Do you regularly visit the dentist for routine dental care? □ Yes □ No

## Section C: Desired aesthetic treatments and shade preferences

1. **Do you feel you have sufficient knowledge about common aesthetic dental treatments (e.g., whitening, veneers, and aligners)?**

□ Yes

□ No

1. **If you required a posterior restoration, which material would you prefer for yourself?**

□ Tooth-colored (composite)

□ Amalgam

□ Glass Ionomer/ Bioactive

1. **Which aesthetic dental treatment would you personally consider? (Choose one)**
   □ Teeth whitening
   □ Orthodontic treatment
   □ Tooth-colored restorations (composite)
   □ Ceramic veneers
   □ Crowns
   □ Implants

□ Partial dentures

□ I do not need treatment

1. **Which tooth shade do you consider most aesthetic for anterior teeth?**
   □ Natural white (A1–A2)
   □ Bright white (B1–BL1)
   □ Moderate white (A3–B2)
   □ Darker/yellowish (A3.5–B3)
   □ I have no preference

## Section D: Influence of Social Media on Aesthetic Perception

**1. Which ONE platform do you mainly use to view dental/aesthetic content?**
□ Instagram

□ TikTok

□ YouTube

□ Snapchat

□ Facebook

□ X (Twitter)

□ I do not follow dental/aesthetic content

**2. How often do you personally view content related to dental aesthetics (whitening, veneers, smile makeovers, aligners, etc.) on social media?**

□ Never

□ Rarely

□ Sometimes

□ Often

□ Very often

**3. To what extent does social media influence your idea of an ideal smile?**
□ Not at all

□ Slightly

□ Moderately

□ Strongly

□ Very strongly

**4. Have you ever considered undergoing an aesthetic dental procedure (e.g., whitening, veneers, aligners) because of something you saw on social media?**
□ Yes

□ No

**5. Have you ever searched online for information, tutorials, or courses on aesthetic dentistry because of social media content?**□ Yes

□ No

**6. Under routine clinical practice conditions, do you believe the aesthetic results shown on social media are clinically achievable (i.e., realistic to reproduce in practice)?**
□ Yes

□ No

**7. To what extent do social-media trends affect your own expectations about dental appearance (e.g., tooth shade, alignment, symmetry)?**

□ Not at all

□ Slightly

□ Moderately

□ Greatly

□ Extremely

**8. Would you like aesthetic dentistry to be more emphasized in your dental education because of what you see on social media?**
□ Yes

□ No

9. **How trustworthy do you consider dental aesthetic content shared by social-media influencers (dentists or non-dentists)?**

□ Not at all

□ Slightly

□ Moderately

□ Very

□ Extremely

**Section E: Professional Aspects of Dental Aesthetics**

**1. Do you believe a dentist’s own smile and facial appearance influence patient trust and acceptance of treatment?**□ Yes

□ No

2. **Would you personally consider undergoing an aesthetic dental procedure (e.g., whitening, alignment, veneers) before starting clinical practice in order to enhance your professional image?**□ Yes

□ No

**3. How often do friends, family, or peers show you photos or social-media examples of ideal smiles and ask for your opinion as a dental student?**

□ Never

□ Rarely

□ Sometimes

□ Often

□ Very often

**4. Do you feel confident explaining the difference between realistic clinical outcomes and digitally altered or filtered smile images shown on social media?**□ Yes

□ No

**5. To what extent does dissatisfaction with your own smile affect your social, academic, or professional confidence?**□ Not at all

□ Slightly

□ Moderately

□ Strongly

□ Very strongly

**6. If cost were not a barrier, would you undergo aesthetic dental treatment?**

□ Yes

□ No
