# Supplementary material for: Self-perception of dental aesthetics and social media influence among students at a Palestinian dental school
Source: BDJ Open. 2026 May 19;12:53. doi: 10.1038/s41405-026-00445-w (PMC13184236; doi:10.1038/s41405-026-00445-w)
Supplement: Supplementary file 2 — Appendix 2 [file 41405_2026_445_MOESM2_ESM.docx]

| Appendix 2: Appendix 2. Section B–E item responses by academic year and gender (counts) | | | | | | | | | | | |
| --- | --- | --- | --- | --- | --- | --- | --- | --- | --- | --- | --- |
|  |  | Males | | | | | Females | | | | |
| Question (item) | Answer | M-1st Year | M-2nd Year | M-3rd Year | M-4th Year | M-5th Year | F-1st Year | F-2nd Year | F-3rd Year | F-4th Year | F-5th Year |
| B1. Prefer selfies from a particular side | Yes | 1 | 3 | 13 | 10 | 19 | 17 | 18 | 21 | 30 | 54 |
|  | No | 2 | 1 | 3 | 8 | 7 | 3 | 6 | 5 | 9 | 16 |
| B2. Wish smile looked like those in media | Yes | 1 | 2 | 6 | 13 | 9 | 12 | 13 | 13 | 16 | 34 |
|  | No | 2 | 2 | 10 | 5 | 17 | 8 | 11 | 13 | 23 | 36 |
| B3. Pay particular attention to others’ teeth/smile | Yes | 3 | 3 | 13 | 18 | 24 | 17 | 23 | 26 | 38 | 69 |
|  | No | 0 | 1 | 3 | 0 | 2 | 3 | 1 | 0 | 1 | 1 |
| B4. Notice gum defects when smiling in mirror | Yes | 1 | 3 | 1 | 13 | 19 | 14 | 10 | 18 | 18 | 49 |
|  | No | 2 | 1 | 15 | 5 | 7 | 6 | 14 | 8 | 21 | 21 |
| B5. Notice tooth defects when smiling in mirror | Yes | 1 | 0 | 1 | 4 | 2 | 0 | 6 | 4 | 9 | 15 |
|  | No | 2 | 4 | 15 | 14 | 24 | 20 | 18 | 22 | 30 | 55 |
| B6. Satisfied with tooth color | Yes | 1 | 2 | 12 | 10 | 18 | 11 | 15 | 18 | 27 | 51 |
|  | No | 2 | 2 | 4 | 8 | 8 | 9 | 9 | 8 | 12 | 19 |
| B7. Satisfied with gum appearance | Yes | 2 | 3 | 16 | 13 | 24 | 20 | 20 | 22 | 34 | 59 |
|  | No | 1 | 1 | 0 | 5 | 2 | 0 | 4 | 4 | 5 | 11 |
| B8. Tooth display in smile is not ideal | Yes | 1 | 0 | 0 | 4 | 7 | 4 | 3 | 4 | 8 | 22 |
|  | No | 2 | 4 | 16 | 14 | 19 | 16 | 21 | 22 | 31 | 48 |
| B9. Dissatisfied with amount of gum show | Yes | 1 | 0 | 0 | 1 | 4 | 1 | 6 | 7 | 7 | 21 |
|  | No | 2 | 4 | 16 | 17 | 22 | 19 | 18 | 19 | 32 | 49 |
| C1. Sufficient knowledge about common aesthetic treatments | Yes | 1 | 1 | 12 | 8 | 18 | 8 | 15 | 9 | 22 | 42 |
|  | No | 2 | 3 | 4 | 10 | 8 | 12 | 9 | 17 | 17 | 28 |
| C2. Preferred material for a posterior restoration | Tooth-coloured (composite) | 2 | 1 | 14 | 16 | 24 | 12 | 17 | 25 | 37 | 67 |
|  | Amalgam | 1 | 3 | 2 | 2 | 1 | 4 | 5 | 1 | 0 | 2 |
|  | Glass ionomer/Bioactive | 0 | 0 | 0 | 0 | 1 | 4 | 2 | 0 | 2 | 1 |
| C3. Aesthetic treatment personally preferred | Teeth whitening | 2 | 2 | 7 | 5 | 9 | 5 | 12 | 11 | 16 | 30 |
|  | Orthodontic treatment | 0 | 1 | 0 | 8 | 5 | 9 | 3 | 10 | 8 | 15 |
|  | Tooth-colored restorations (composite) | 0 | 0 | 0 | 2 | 1 | 0 | 0 | 0 | 0 | 5 |
|  | Ceramic veneers | 0 | 1 | 3 | 0 | 6 | 0 | 2 | 3 | 3 | 3 |
|  | Crowns | 0 | 0 | 0 | 0 | 1 | 1 | 0 | 0 | 0 | 0 |
|  | Implants | 0 | 0 | 0 | 2 | 0 | 0 | 0 | 0 | 1 | 0 |
|  | Partial dentures | 0 | 0 | 0 | 0 | 0 | 0 | 0 | 0 | 0 | 0 |
|  | I do not need treatment | 1 | 0 | 6 | 1 | 4 | 5 | 7 | 2 | 11 | 17 |
| C4. Most aesthetic shade for anterior teeth | Natural white (A1–A2) | 2 | 1 | 11 | 17 | 19 | 10 | 19 | 17 | 36 | 63 |
|  | Bright white (B1–BL1) | 0 | 0 | 2 | 0 | 1 | 0 | 1 | 1 | 1 | 2 |
|  | Moderate white (A3–B2) | 1 | 2 | 1 | 1 | 5 | 10 | 4 | 5 | 2 | 4 |
|  | Darker/yellowish (A3.5–B3) | 0 | 0 | 1 | 0 | 0 | 0 | 0 | 1 | 0 | 0 |
|  | No preference | 0 | 1 | 1 | 0 | 1 | 0 | 0 | 2 | 0 | 1 |
| D1. Main platform used to view dental/aesthetic content | Instagram | 2 | 3 | 15 | 14 | 19 | 14 | 20 | 21 | 35 | 55 |
|  | TikTok | 0 | 1 | 1 | 2 | 2 | 3 | 3 | 5 | 2 | 8 |
|  | YouTube | 1 | 0 | 0 | 2 | 4 | 0 | 1 | 0 | 1 | 1 |
|  | Snapchat | 0 | 0 | 0 | 0 | 0 | 0 | 0 | 0 | 0 | 0 |
|  | Facebook | 0 | 0 | 0 | 0 | 1 | 1 | 0 | 0 | 1 | 6 |
|  | X (Twitter) | 0 | 0 | 0 | 0 | 0 | 0 | 0 | 0 | 0 | 0 |
|  | Other/None | 0 | 0 | 0 | 0 | 0 | 2 | 0 | 0 | 0 | 0 |
| D2. Frequency of viewing dental/aesthetic content | Never | 0 | 0 | 0 | 0 | 0 | 1 | 0 | 0 | 1 | 0 |
|  | Rarely | 0 | 1 | 2 | 1 | 1 | 3 | 1 | 2 | 0 | 2 |
|  | Sometimes | 3 | 3 | 8 | 10 | 8 | 9 | 13 | 13 | 14 | 29 |
|  | Often | 0 | 0 | 2 | 4 | 12 | 6 | 9 | 9 | 15 | 30 |
|  | Very often | 0 | 0 | 4 | 3 | 5 | 1 | 1 | 2 | 9 | 9 |
| D3. Social media influence on the idea of an ideal smile | Not at all | 0 | 0 | 5 | 2 | 2 | 0 | 2 | 3 | 4 | 9 |
|  | Slightly | 0 | 3 | 1 | 2 | 4 | 4 | 5 | 3 | 4 | 19 |
|  | Moderately | 2 | 1 | 2 | 10 | 10 | 8 | 10 | 12 | 16 | 24 |
|  | Strongly | 0 | 0 | 3 | 4 | 10 | 5 | 7 | 7 | 14 | 15 |
|  | Very strongly | 1 | 0 | 5 | 0 | 0 | 3 | 0 | 1 | 1 | 3 |
| D4. Considered an aesthetic procedure due to social media | Yes | 1 | 3 | 6 | 8 | 14 | 6 | 12 | 12 | 21 | 33 |
|  | No | 2 | 1 | 10 | 10 | 12 | 14 | 12 | 14 | 18 | 37 |
| D5. Searched online for information/tutorials due to online content | Yes | 1 | 1 | 5 | 7 | 19 | 8 | 15 | 11 | 23 | 40 |
|  | No | 2 | 3 | 11 | 11 | 7 | 12 | 9 | 15 | 16 | 30 |
| D6. Perceived feasibility of social-media aesthetic outcomes under routine clinical practice conditions | Yes | 1 | 3 | 13 | 13 | 16 | 14 | 22 | 19 | 25 | 31 |
|  | No | 2 | 1 | 3 | 5 | 10 | 6 | 2 | 7 | 14 | 39 |
| D7. Social media trends affect personal esthetic expectations | Not at all | 0 | 0 | 2 | 1 | 4 | 3 | 3 | 1 | 7 | 8 |
|  | Slightly | 0 | 2 | 3 | 3 | 9 | 1 | 4 | 4 | 8 | 18 |
|  | Moderately | 1 | 2 | 6 | 7 | 4 | 12 | 12 | 13 | 10 | 27 |
|  | Greatly | 1 | 0 | 4 | 6 | 6 | 2 | 4 | 7 | 12 | 15 |
|  | Extremely | 1 | 0 | 1 | 1 | 3 | 2 | 1 | 1 | 2 | 2 |
| D8. Want more aesthetic dentistry emphasis in education | Yes | 2 | 4 | 13 | 10 | 21 | 15 | 22 | 25 | 31 | 59 |
|  | No | 1 | 0 | 3 | 8 | 5 | 5 | 2 | 1 | 8 | 11 |
| D9. Trustworthiness of social media aesthetic dental content | Not at all | 0 | 0 | 1 | 1 | 0 | 0 | 0 | 0 | 1 | 1 |
|  | Slightly | 1 | 0 | 1 | 5 | 6 | 4 | 2 | 4 | 9 | 17 |
|  | Moderately | 1 | 3 | 11 | 8 | 17 | 13 | 15 | 18 | 21 | 45 |
|  | Very | 1 | 1 | 1 | 4 | 2 | 3 | 7 | 4 | 6 | 7 |
|  | Extremely | 0 | 0 | 2 | 0 | 1 | 0 | 0 | 0 | 2 | 0 |
| E1. Dentist’s smile influences patient trust | Yes | 3 | 4 | 15 | 16 | 26 | 20 | 23 | 26 | 38 | 67 |
|  | No | 0 | 0 | 1 | 2 | 0 | 0 | 1 | 0 | 1 | 3 |
| E2. Consider aesthetic treatment to enhance professional image | Yes | 2 | 4 | 7 | 10 | 14 | 11 | 13 | 18 | 19 | 41 |
|  | No | 1 | 0 | 9 | 8 | 12 | 9 | 11 | 8 | 20 | 29 |
| E3. How often others ask your opinion on “ideal smiles” | Never | 1 | 0 | 3 | 2 | 2 | 4 | 4 | 4 | 5 | 3 |
|  | Rarely | 0 | 1 | 4 | 4 | 1 | 3 | 7 | 3 | 5 | 12 |
|  | Sometimes | 1 | 2 | 6 | 6 | 10 | 10 | 11 | 14 | 23 | 31 |
|  | Often | 1 | 0 | 0 | 5 | 10 | 2 | 2 | 5 | 3 | 17 |
|  | Very often | 0 | 1 | 3 | 1 | 3 | 1 | 0 | 0 | 3 | 7 |
| E4. Confident explaining feasible outcomes vs enhanced presentation | Yes | 1 | 2 | 9 | 11 | 24 | 12 | 18 | 17 | 30 | 52 |
|  | No | 2 | 2 | 7 | 7 | 2 | 8 | 6 | 9 | 9 | 18 |
| E5. Extent dissatisfaction affects social/academic/professional confidence | Not at all | 0 | 1 | 10 | 10 | 13 | 5 | 12 | 11 | 19 | 28 |
|  | Slightly | 1 | 1 | 2 | 4 | 6 | 7 | 6 | 6 | 9 | 21 |
|  | Moderately | 2 | 2 | 2 | 3 | 4 | 5 | 4 | 4 | 7 | 11 |
|  | Strongly | 0 | 0 | 0 | 0 | 1 | 1 | 2 | 4 | 2 | 8 |
|  | Very strongly | 0 | 0 | 2 | 1 | 2 | 2 | 0 | 1 | 2 | 2 |
| E6. Would undergo aesthetic treatment if cost not a barrier | Yes | 3 | 2 | 2 | 9 | 11 | 12 | 10 | 13 | 17 | 37 |
|  | No | 0 | 2 | 14 | 9 | 15 | 8 | 14 | 13 | 22 | 33 |
