# Supplementary material for: Self-perception of dental aesthetics and social media influence among students at a Palestinian dental school
Source: BDJ Open. 2026 May 19;12:53. doi: 10.1038/s41405-026-00445-w (PMC13184236; doi:10.1038/s41405-026-00445-w)
Supplement: Supplementary file 3 — Appendix 3 [file 41405_2026_445_MOESM3_ESM.docx]

**Appendix 3. Regression Models (Recomputed from Raw Datasheet)**

All models were rerun from the uploaded raw datasheet (n = 246) using binary logistic regression (enter method) with two covariates: gender (Female vs Male) and training stage (Clinical vs Preclinical). Odds ratios are adjusted odds ratios (aOR) with 95% confidence intervals (CI). For multinomial models, relative risk ratios (RRR) are reported with Whitening as the reference category.

## Appendix Table B. Section B – Logistic regression outputs for all items

| **Outcome** | **Event coded** | **Predictor (reference)** | **β** | **SE** | **aOR** | **95% CI** | **p value** |
| --- | --- | --- | --- | --- | --- | --- | --- |
| B1. Prefer taking selfies from a particular side (because smile looks better) | Yes | Female vs Male (Male) | 0.484 | 0.32 | 1.62 | 0.87–3.04 | 0.131 |
| B1. Prefer taking selfies from a particular side (because smile looks better) | Yes | Clinical vs Preclinical (Preclinical) | -0.238 | 0.314 | 0.79 | 0.43–1.46 | 0.449 |
| B2. Wish smile looked like those in media | Yes | Female vs Male (Male) | 0.11 | 0.287 | 1.12 | 0.64–1.96 | 0.703 |
| B2. Wish smile looked like those in media | Yes | Clinical vs Preclinical (Preclinical) | -0.135 | 0.263 | 0.87 | 0.52–1.46 | 0.608 |
| B3. Pay particular attention to others’ teeth/smile | Yes | Female vs Male (Male) | 1.135 | 0.607 | 3.11 | 0.95–10.22 | 0.061 |
| B3. Pay particular attention to others’ teeth/smile | Yes | Clinical vs Preclinical (Preclinical) | 1.33 | 0.635 | 3.78 | 1.09–13.11 | 0.036 |
| B4. Notice gum defects when smiling in mirror | Yes | Female vs Male (Male) | 0.267 | 0.293 | 1.31 | 0.74–2.32 | 0.362 |
| B4. Notice gum defects when smiling in mirror | Yes | Clinical vs Preclinical (Preclinical) | 0.598 | 0.269 | 1.82 | 1.07–3.08 | 0.026 |
| B5. Notice tooth defects when smiling in mirror | Yes | Female vs Male (Male) | 0.575 | 0.424 | 1.78 | 0.77–4.08 | 0.175 |
| B5. Notice tooth defects when smiling in mirror | Yes | Clinical vs Preclinical (Preclinical) | 0.523 | 0.372 | 1.69 | 0.81–3.50 | 0.16 |
| B6. Satisfied with tooth color | Yes | Female vs Male (Male) | 0.192 | 0.302 | 1.21 | 0.67–2.19 | 0.526 |
| B6. Satisfied with tooth color | Yes | Clinical vs Preclinical (Preclinical) | 0.27 | 0.278 | 1.31 | 0.76–2.26 | 0.331 |
| B7. Satisfied with gum appearance | Yes | Female vs Male (Male) | -0.015 | 0.421 | 0.98 | 0.43–2.25 | 0.971 |
| B7. Satisfied with gum appearance | Yes | Clinical vs Preclinical (Preclinical) | -0.385 | 0.404 | 0.68 | 0.31–1.50 | 0.341 |
| B8. Tooth display in smile is not ideal | Yes | Female vs Male (Male) | 0.357 | 0.37 | 1.43 | 0.69–2.95 | 0.334 |
| B8. Tooth display in smile is not ideal | Yes | Clinical vs Preclinical (Preclinical) | 0.921 | 0.36 | 2.51 | 1.24–5.09 | 0.011 |
| B9. Dissatisfied with amount of gum show | Yes | Female vs Male (Male) | 1.161 | 0.464 | 3.19 | 1.29–7.93 | 0.012 |
| B9. Dissatisfied with amount of gum show | Yes | Clinical vs Preclinical (Preclinical) | 0.406 | 0.348 | 1.5 | 0.76–2.97 | 0.244 |
| B10. Feel teeth are too long or too short | Yes | Female vs Male (Male) | 0.348 | 0.432 | 1.42 | 0.61–3.30 | 0.421 |
| B10. Feel teeth are too long or too short | Yes | Clinical vs Preclinical (Preclinical) | 0.708 | 0.411 | 2.03 | 0.91–4.54 | 0.085 |
| B11. Feel teeth are too wide or too narrow | Yes | Female vs Male (Male) | 0.311 | 0.43 | 1.36 | 0.59–3.17 | 0.469 |
| B11. Feel teeth are too wide or too narrow | Yes | Clinical vs Preclinical (Preclinical) | -0.042 | 0.371 | 0.96 | 0.46–1.98 | 0.91 |
| B12. Satisfied with shape of teeth | Yes | Female vs Male (Male) | -0.26 | 0.38 | 0.77 | 0.37–1.62 | 0.494 |
| B12. Satisfied with shape of teeth | Yes | Clinical vs Preclinical (Preclinical) | 0.511 | 0.325 | 1.67 | 0.88–3.15 | 0.116 |
| B13. Regularly visit the dentist | Yes | Female vs Male (Male) | 0.433 | 0.29 | 1.54 | 0.87–2.72 | 0.135 |
| B13. Regularly visit the dentist | Yes | Clinical vs Preclinical (Preclinical) | 0.432 | 0.266 | 1.54 | 0.91–2.59 | 0.105 |

Notes (B): Event coded denotes outcome = 1 (Yes), except where specified. Reference categories were Male and Preclinical. Two-tailed p < 0.05 considered statistically significant.

## Appendix Table C1. Section C – Binary logistic regression models

| **Outcome** | **Event coded** | **Predictor (reference)** | **β** | **SE** | **aOR** | **95% CI** | **p value** |
| --- | --- | --- | --- | --- | --- | --- | --- |
| C1. Knowledge of aesthetic treatments | Yes | Female vs Male (Male) | -0.232 | 0.292 | 0.79 | 0.45–1.41 | 0.428 |
| C1. Knowledge of aesthetic treatments | Yes | Clinical vs Preclinical (Preclinical) | 0.37 | 0.265 | 1.45 | 0.86–2.43 | 0.163 |
| C2. Posterior material preference (Composite vs Other) | Yes | Female vs Male (Male) | 0.385 | 0.432 | 1.47 | 0.63–3.43 | 0.373 |
| C2. Posterior material preference (Composite vs Other) | Yes | Clinical vs Preclinical (Preclinical) | 1.623 | 0.423 | 5.07 | 2.21–11.62 | <0.001 |
| C3. Anterior shade preference (A1–A2 vs Other) | Yes | Female vs Male (Male) | 0.485 | 0.358 | 1.62 | 0.81–3.28 | 0.175 |
| C3. Anterior shade preference (A1–A2 vs Other) | Yes | Clinical vs Preclinical (Preclinical) | 1.45 | 0.335 | 4.26 | 2.21–8.21 | <0.001 |

Notes (C1): Section C outcomes were dichotomized as follows: posterior material preference (Composite vs Other), anterior shade preference (A1–A2 vs Other), and knowledge of esthetic treatments (Yes vs No). Reference categories were Male and Preclinical.

## Appendix Table C2. Section C – Multinomial logistic regression model

| **Outcome** | **Comparison vs reference** | **Predictor** | **β** | **SE** | **RRR** | **95% CI** | **p value** |
| --- | --- | --- | --- | --- | --- | --- | --- |
| Desired aesthetic treatment | Orthodontics vs Whitening | Female vs Male | 0.085 | 0.448 | 1.09 | 0.45–2.62 | 0.849 |
| Desired aesthetic treatment | Orthodontics vs Whitening | Clinical vs Preclinical | 0.006 | 0.386 | 1.01 | 0.47–2.15 | 0.987 |
| Desired aesthetic treatment | No treatment vs Whitening | Female vs Male | -1.036 | 0.461 | 0.35 | 0.14–0.88 | 0.025 |
| Desired aesthetic treatment | No treatment vs Whitening | Clinical vs Preclinical | 0.392 | 0.467 | 1.48 | 0.59–3.69 | 0.401 |
| Desired aesthetic treatment | Other treatments vs Whitening | Female vs Male | -0.083 | 0.384 | 0.92 | 0.43–1.95 | 0.829 |
| Desired aesthetic treatment | Other treatments vs Whitening | Clinical vs Preclinical | -0.02 | 0.337 | 0.98 | 0.51–1.90 | 0.954 |

Notes (C2): RRR = relative risk ratio. The outcome reference category was Whitening. Predictors entered simultaneously; reference groups were Male and Preclinical.

## Appendix Table D. Section D – Binary logistic regression models

| **Outcome** | **Event coded** | **Predictor (reference)** | **β** | **SE** | **aOR** | **95% CI** | **p value** |
| --- | --- | --- | --- | --- | --- | --- | --- |
| D1. Main platform Instagram (vs other) | Yes | Female vs Male (Male) | 0.119 | 0.356 | 1.13 | 0.56–2.26 | 0.739 |
| D1. Main platform Instagram (vs other) | Yes | Clinical vs Preclinical (Preclinical) | -0.011 | 0.333 | 0.99 | 0.52–1.90 | 0.973 |
| D2. Viewed aesthetic content ≥ sometimes | Yes | Female vs Male (Male) | 0.383 | 0.576 | 1.47 | 0.47–4.54 | 0.506 |
| D2. Viewed aesthetic content ≥ sometimes | Yes | Clinical vs Preclinical (Preclinical) | 1.291 | 0.566 | 3.63 | 1.20–11.03 | 0.023 |
| D3. Moderate–very strong SM influence | Yes | Female vs Male (Male) | -0.066 | 0.317 | 0.94 | 0.50–1.74 | 0.836 |
| D3. Moderate–very strong SM influence | Yes | Clinical vs Preclinical (Preclinical) | -0.105 | 0.291 | 0.9 | 0.51–1.59 | 0.718 |
| D4. Considered aesthetic procedure due to SM | Yes | Female vs Male (Male) | -0.021 | 0.288 | 0.98 | 0.56–1.72 | 0.942 |
| D4. Considered aesthetic procedure due to SM | Yes | Clinical vs Preclinical (Preclinical) | 0.268 | 0.265 | 1.31 | 0.78–2.20 | 0.312 |
| D5. Searched tutorials due to SM | Yes | Female vs Male (Male) | 0.23 | 0.29 | 1.26 | 0.71–2.22 | 0.428 |
| D5. Searched tutorials due to SM | Yes | Clinical vs Preclinical (Preclinical) | 0.578 | 0.266 | 1.78 | 1.06–3.00 | 0.03 |
| D6. SM outcomes judged not achievable | Not achievable | Female vs Male (Male) | 0.357 | 0.313 | 1.43 | 0.77–2.64 | 0.254 |
| D6. SM outcomes judged not achievable | Not achievable | Clinical vs Preclinical (Preclinical) | 1.028 | 0.298 | 2.8 | 1.56–5.01 | <0.001 |
| D7. SM trends affect expectations ≥ moderate | Yes | Female vs Male (Male) | 0.152 | 0.304 | 1.16 | 0.64–2.11 | 0.616 |
| D7. SM trends affect expectations ≥ moderate | Yes | Clinical vs Preclinical (Preclinical) | -0.614 | 0.293 | 0.54 | 0.30–0.96 | 0.036 |
| D8. Want more aesthetic emphasis in education | Yes | Female vs Male (Male) | 0.63 | 0.352 | 1.88 | 0.94–3.74 | 0.073 |
| D8. Want more aesthetic emphasis in education | Yes | Clinical vs Preclinical (Preclinical) | -0.559 | 0.37 | 0.57 | 0.28–1.18 | 0.131 |
| D9. Trust SM content ≥ moderate | Yes | Female vs Male (Male) | -0.143 | 0.379 | 0.87 | 0.41–1.82 | 0.706 |
| D9. Trust SM content ≥ moderate | Yes | Clinical vs Preclinical (Preclinical) | -0.431 | 0.346 | 0.65 | 0.33–1.28 | 0.213 |

Notes (D): D2 was dichotomized as sometimes/often/very often vs rarely/never. D3, D7, and D9 were dichotomized as ≥ moderate vs lower categories. For D6, the modeled event was Not achievable (coded 1).

## Appendix Table E. Section E – Binary logistic regression models

| **Outcome** | **Event coded** | **Predictor (reference)** | **β** | **SE** | **aOR** | **95% CI** | **p value** |
| --- | --- | --- | --- | --- | --- | --- | --- |
| E1. Dentist smile influences patient trust | Yes | Female vs Male (Male) | 0.464 | 0.746 | 1.59 | 0.37–6.87 | 0.534 |
| E1. Dentist smile influences patient trust | Yes | Clinical vs Preclinical (Preclinical) | -0.599 | 0.828 | 0.55 | 0.11–2.79 | 0.469 |
| E2. Would undergo aesthetic treatment for professional image | Yes | Female vs Male (Male) | 0.063 | 0.289 | 1.07 | 0.60–1.88 | 0.826 |
| E2. Would undergo aesthetic treatment for professional image | Yes | Clinical vs Preclinical (Preclinical) | -0.171 | 0.267 | 0.84 | 0.50–1.42 | 0.522 |
| E6. Would undergo treatment if cost not a barrier | Yes | Female vs Male (Male) | 0.389 | 0.291 | 1.48 | 0.83–2.61 | 0.181 |
| E6. Would undergo treatment if cost not a barrier | Yes | Clinical vs Preclinical (Preclinical) | 0.145 | 0.265 | 1.16 | 0.69–1.94 | 0.584 |
| E3. Asked opinions on ideal smiles ≥ sometimes | Yes | Female vs Male (Male) | -0.02 | 0.326 | 0.98 | 0.52–1.86 | 0.952 |
| E3. Asked opinions on ideal smiles ≥ sometimes | Yes | Clinical vs Preclinical (Preclinical) | 0.701 | 0.29 | 2.02 | 1.14–3.56 | 0.016 |
| E4. Confidence explaining feasible outcomes vs enhanced presentation | Yes | Female vs Male (Male) | 0.127 | 0.318 | 1.14 | 0.61–2.12 | 0.69 |
| E4. Confidence explaining feasible outcomes vs enhanced presentation | Yes | Clinical vs Preclinical (Preclinical) | 0.633 | 0.288 | 1.88 | 1.07–3.31 | 0.028 |

Notes (E): E3 was dichotomized as ≥ sometimes vs never/rarely. Reference categories were Male and Preclinical.
